# Targeted therapy and immunotherapy: Diamonds in the rough in the treatment of epithelial ovarian cancer

**DOI:** 10.3389/fphar.2023.1131342

**Published:** 2023-03-24

**Authors:** Xu Huang, Xiao-Yu Li, Wu-Lin Shan, Yao Chen, Qi Zhu, Bai-Rong Xia

**Affiliations:** ^1^ The First Affiliated Hospital of USTC, Division of Life Sciences and Medicine, University of Science and Technology of China, Hefei, Anhui, China; ^2^ Bengbu Medical College Bengbu, Anhui, China; ^3^ Department of Obstetrics and Gynecology, The First Affiliated Hospital of USTC, Division of Life Sciences and Medicine, University of Science and Technology of China, Anhui Provincial Cancer Hospital, Hefei, Anhui, China

**Keywords:** epithelial ovarian cancer, angiogenesis inhibitor, poly (ADP ribose) polymerase inhibitor, immunotherapy, targeted therapy, tumor microenvironment, clinical trials

## Abstract

Currently, for ovarian cancer, which has the highest mortality rate among all gynecological cancers, the standard treatment protocol is initial tumor cytoreductive surgery followed by platinum-based combination chemotherapy. Although the survival rate after standard treatment has improved, the therapeutic effect of traditional chemotherapy is very limited due to problems such as resistance to platinum-based drugs and recurrence. With the advent of the precision medicine era, molecular targeted therapy has gradually entered clinicians’ view, and individualized precision therapy has been realized, surpassing the limitations of traditional therapy. The detection of genetic mutations affecting treatment, especially breast cancer susceptibility gene (BRCA) mutations and mutations of other homologous recombination repair defect (HRD) genes, can guide the targeted drug treatment of patients, effectively improve the treatment effect and achieve a better patient prognosis. This article reviews different sites and pathways of targeted therapy, including angiogenesis, cell cycle and DNA repair, and immune and metabolic pathways, and the latest research progress from preclinical and clinical trials related to ovarian cancer therapy.

## 1 Introduction

Among cancers of the female reproductive system, ovarian cancer (OC) ranks first in terms of recurrence, morbidity and mortality (Lheureux et al., 2019a) and is a serious threat to women’s health. According to the survey statistics of the American Cancer Society, there will be 19,880 new cases of OC and 12,810 deaths in the United States in 2022 (Siegel et al., 2022). Approximately 85%–90% of OCs are epithelial in nature (Sisay and Edessa, 2017); however, due to the lack of obvious symptoms in the early stages of epithelial ovarian cancer (EOC) and the lack of effective early screening tools, the EOC of patients is already at an advanced stage (stage III-IV) at the time of diagnosis (Miller et al., 2020). Timely tumor cytoreduction combined with platinum-based chemotherapy combined/not combined with targeted maintenance therapy has become the initial standard of care for OC (Buechel et al., 2019) but tumor recurrence or persistence, with a median progression-free survival (mPFS) of only 12–18 months (Boussios et al., 2020) and ultimately no treatment, resulting in a 5-year overall survival (OS) rate of only approximately 30% (Lheureux et al., 2019b). Accordingly, a significant need for improved therapeutic approaches more importance has been attached to cancer biological research, which aids the discovery of novel biomarkers, defining more effective molecular targets, and developing new treatment strategies.

**FIGURE 1:**
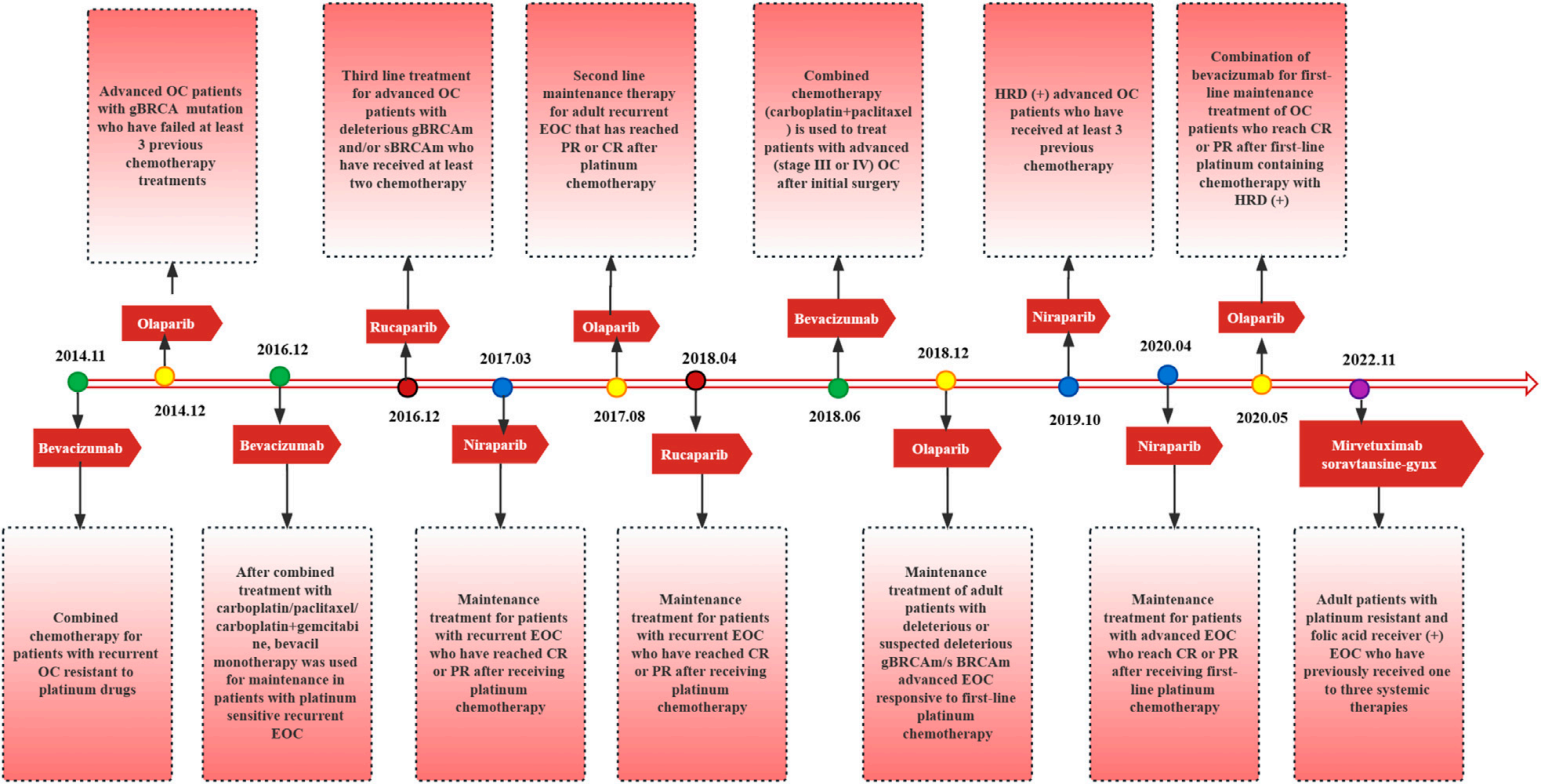
Targeted drugs approved by FDA, approval time and corresponding indications.

Targeted therapies and immunotherapies have emerged as novel treatment strategies for ovarian cancer, which driven the management of ovarian cancer into individualized treatments. A drug targeting angiogenesis, bevacizumab, combined with platinum/taxane-based chemotherapy prolongs progression-free survival (PFS) by 3.5 months in patients with OC and has been recommended by National Comprehensive Cancer Network (NCCN) guidelines as a first-line treatment for OC (Armstrong et al., 2022). In addition, approximately 30% of epithelial ovarian tumors have homologous recombination repair defects (HRDs). EOC tumors with HRDs are resistant to platinum-based chemotherapy, and these tumors show higher sensitivity to poly (ADP ribose) polymerase inhibitor (PARPi) therapy (Miller et al., 2020; Vergote et al., 2022). PARPis have been widely used for first-line maintenance treatment (Lorusso et al., 2020) and second-line and beyond treatment of OC, significantly improving patient (Figure 1) (DiSilvestro and Alvarez Secord, 2018). Currently, the main challenge facing PARPis in clinical application is drug resistance (Li et al., 2020). However, due to the immunosuppressive tumor microenvironment (TME) of OC, monotherapy with immune checkpoint inhibitors (ICIs) targeting PD-1/PD-L1 and CTLA-4 has not achieved therapeutic effects to the satisfaction of investigators when compared to the effects of targeted agents (Kandalaft et al., 2019). Therefore, focusing on the application of ICIs in combination with chemotherapy and targeted therapy to explore a treatment strategy of one plus one over two is a meaningful research direction for the future (Yang et al., 2020).

In this review, we aimed to discuss the application of targeted lipid metabolism therapies based on omental metastatic OC, as well as immunotherapies other than targeted ICIs (Odunsi, 2017), such as immunization vaccines and oncolytic virus therapy, in the hope of providing possible strategies for the future treatment of EOC (Ventriglia et al., 2017). We also reviewed the results and implications of trials evaluating therapy and immunotherapy in the front-line setting to define the optimal positioning of these agents in the treatment for ovarian cancer and provide a focus on preclinical studies and ongoing clinical trials of combined targeted therapy and immunotherapy as well as perspectives and potential challenges of this combination strategy.

## 2 Targeting angiogenesis

**FIGURE 2:**
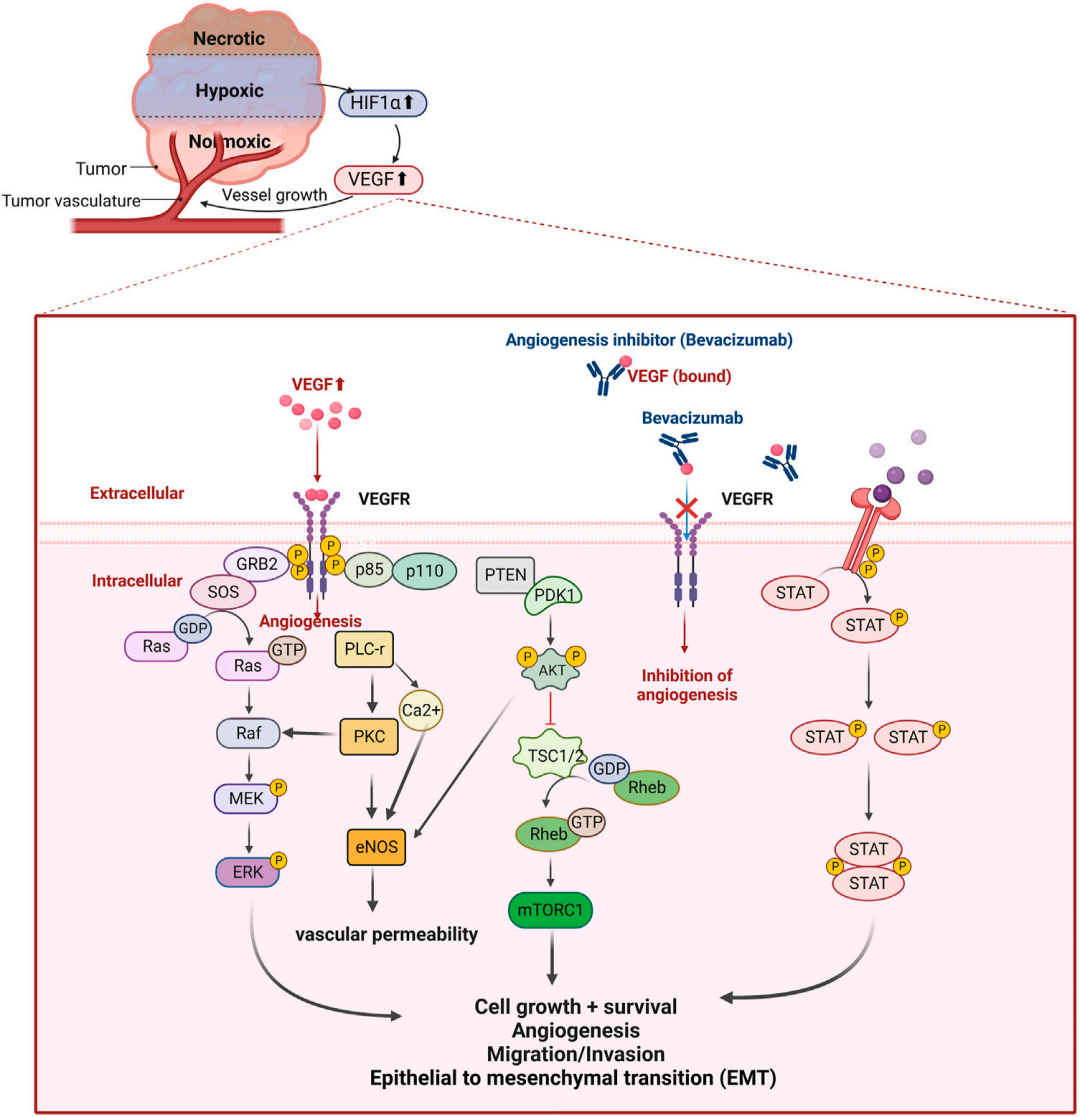
Hypoxic microenvironment in ovarian cancer and the principle of action of angiogenesis inhibitors. The hypoxic microenvironment inside the tumor mass induces increased HIF-1α expression and upregulates vascular endothelial growth factor (VEGF), which regulates tumor angiogenesis by binding to its receptor and activates intracellular signaling pathways. Expression to promote EMT-induced angiogenesis mimicry; and activation of JAK-STAT signaling pathway to participate in angiogenesis. Bevacizumab, a recombinant humanized monoclonal antibody targeting VEGF, inhibits tumor neovascularization by specifically binding to VEGF and preventing its binding to VEGFR, blocking the signaling pathway of angiogenesis.

The recurrence and metastasis of EOC mainly manifest in the formation and invasion of abnormal tumor cells and blood vessels, accompanied by chemotherapy drug resistance (Ferrara et al., 2004). Tumor angiogenesis and metastasis involve the overexpression of hypoxia inducible factor (HIF) induced by the hypoxic microenvironment in which tumor cells live (Muz et al., 2015), which further induces the transcription and translation of vascular endothelial growth factors (VEGF) protein (Semenza, 2010) (Figure 2). Tumor cells overexpress VEGF-A, and the upregulated VEGF-A combines with its receptors on the vascular endothelial cell membrane (VEGFR-1 and VEGFR-2) to form a complex, which transmits activation signals to the cascade reaction mediated by mitogen activated protein (MAP) kinase and PI3K/AKT/mTOR, inhibits proapoptotic proteins, leads to cell survival, mediates angiogenesis and lymph angiogenesis, and increases vascular permeability (Aziz et al., 2018; Li et al., 2022c). In preclinical models, VEGF-A signal blockade inhibits angiogenesis and tumor growth, and the new tumor vascular system is particularly sensitive to VEGF-A deprivation (Chen et al., 2019). In this review, we will introduce bevacizumab, which has been approved for use in the treatment of OC, and several potential angiogenesis inhibitors in clinical trials.

### 2.1 Bevacizumab (Avastin)

The binding of bevacizumab to circulating VEGF-A competitively inhibits VEGF-A binding to its endothelial cell surface receptors, ultimately inhibiting abnormal tumor angiogenesis (Figure 2). The 2022 NCCN guidelines recommended the simultaneous addition of bevacizumab to chemotherapy regimens for first-line treatment of OC, platinum-sensitive relapse, and second-line treatment of platinum-resistant relapse and, if effective, bevacizumab maintenance therapy at the end of chemotherapy (Armstrong et al., 2022). Two classic phase III clinical trials, ICON7 and GOG-0218 (Table 1), provided evidence that the addition of bevacizumab to standard first-line chemotherapy for EOC significantly improves PFS, and patients with poor prognosis, such as those with tumors with a high KELIM score and poor chemotherapy sensitivity, can benefit in terms of OS (Burger et al., 2011; Perren et al., 2011). Furthermore, in GOG-218, the analysis of many tumor biomarkers showed a positive correlation between OS and PFS and the efficacy of bevacizumab in first-line chemotherapy as high microvessel density (above the median) increased; similarly, high expression of tVEGF-a was positively associated with prolonged OS (Bais et al., 2017). These findings suggest that in future studies, we could consider microvessel density and tVEGF-a as potential biomarkers to predict the response to first-line treatment with bevacizumab and that specific subgroups of patients with high levels of these biomarkers would be more likely to benefit from first-line treatment including bevacizumab (Bais et al., 2017). Furthermore, in the second-line treatment of patients with platinum-sensitive recurrent OC (PSROC), the OCEANS trial (Table 1) showed that for patients not previously treated with bevacizumab, the mPFS was prolonged by 4 months with the addition of bevacizumab to the carboplatin and gemcitabine (GC) regimen compared to with the GC regimen alone (12.4 months vs 8.4 months), and the efficacy rate (79% vs 57%) indicated that the bevacizumab combination was more effective than the GC regimen alone (Aghajanian et al., 2012). Another trial (NCT01802749) found that bevacizumab remained effective when reintroduced in second-line therapy, with a 3-month prolongation of the mPFS (11.8 months vs 8.8 months). Combining the findings of these two studies, it can be concluded that patients with platinum-sensitive OC benefit from the use of bevacizumab in combination with chemotherapy in second-line therapy regardless of whether bevacizumab is used first and that the amplification of resistant clones may not lead to bevacizumab resistance (Pignata et al., 2021). Furthermore, in the GOG-0213 trial, the mPFS (13.8 months vs 10.4 months) and OS (42.2 months vs 37.3 months) were longer with the paclitaxel and carboplatin (PC) regimen plus bevacizumab than with the PC regimen alone; the effectiveness (78% vs 59%) suggests that the advantage of combining bevacizumab (Coleman et al., 2017). Second, in patients with platinum-resistant OC, AURELIA (Table 1) showed that bevacizumab combined with standard monotherapy was also effective in prolonging PFS (3.4 months vs 6.7 months) and the objective response rate ORR (11.8% vs 27.3%, *p* < 0.01) (Pujade-Lauraine et al., 2014). Intriguingly, a subgroup of patients with malignant ascites was distinguished in the AURELIA trial, and the addition of bevacizumab to chemotherapy was also found to improve ascites control (Pujade-Lauraine et al., 2014). In a GOG-218 subgroup analysis, PFS and OS were prolonged in patients with ascites on bevacizumab, although it was not directly reported whether bevacizumab had a direct effect on ascites control (Ferriss et al., 2015). A phase II clinical trial, REZOLVE, demonstrated the potential of intraperitoneal injection of bevacizumab (IP-bev) in delaying malignant ascites formation in chemotherapy-resistant EOC, and we expect more studies to demonstrate that similar palliative therapies can benefit patients with advanced OC with peritoneal metastases (Sjoquist et al., 2021). In Table 1, we summarize a portion of the phase III clinical trials of bevacizumab for OC to date.

**TABLE 1:** Summary of bevacizumab phase III clinical trial.

Study	Setting	N	Treatment arm	PFS (median, months)	PFS, HR (95% CI)	OS (median, months)	OS, HR (95% CI)	Ref
NCT01239732 **ROSiA**	Stage IIB to IV or Grade 3 Stage I to IIA OC	1,021	Bevacizumab + paclitaxel + carboplatin	25.5 (23.7 to 27.6)	-	-	-	Oza et al. (2017)
NCT00976911 **AURELIA**	Patients with platinum-resistant EOC	361	Ⅰ: Paclitaxel/topotecan/liposomal doxorubicin	3.4 (2.10 to 3.75)	-	13.3 (11.89 to 16.43)	-	Pujade-Lauraine et al. (2014)
			Ⅱ: Paclitaxel/topotecan/liposomal doxorubicin + bevacizumab	6.8 (5.62 to 7.79)	0.48 (0.38 to 0.60, *p* < 0.001)	16.6 (13.70 to 18.99)	0.85 (0.66 to 1.08, *p* = 0.174)	
NCT00434642 **OCEANS**	Patients with platinum-sensitive recurrent OC	484	Ⅰ: Carboplatin + gemcitabine + bevacizumab	12.4 (11.40 to 12.71)	0.484 (0.388 to 0.605, *p* < 0.0001)	33.6 (30.32 to 35.84)	0.952 (0.771 to 1.176, *p* = 0.65)	Aghajanian et al. (2015)
			Ⅱ: Carboplatin + gemcitabine + placebo	8.4 (8.31 to 9.66)	-	32.9 (29.80 to 37.68)	-	
NCT00951496 **GOG-252**	Stage II-III EOC	1,560	Ⅰ: Paclitaxel, IV + bevacizumab, IV + carboplatin, IV	24.9 (22.3 to 27.2)	-	75.4 (67.1 to NA)	-	(Walker et al. (2019))
			Ⅱ: Paclitaxel, IV + bevacizumab, IV + carboplatin, IP	27.4 (24.6 to 28.8)	0.94 (0.81 to 1.09)	74.2 (61.9 to 78.4)	-	
			III: Paclitaxel, IP + bevacizumab, IV + carboplatin, IP	26.2 (23.8 to 28.0)	0.99 (0.86 to 1.15)	67.6 (63.5 to 74.6)	-	
NCT00262847 **GOG-0218**	Newly diagnosed, untreated stage III or IV EOC	1873	Ⅰ: Placebo + paclitaxel + carboplatin	10.3	-	39.3	-	
			Ⅱ: Paclitaxel + carboplatin + bevacizumab throughout	14.1	0.717 (0.625 to 0.824, *p* < 0.001)	39.7	0.915 (0.727 to 1.152, *p* = 0.45)	Burger et al. (2011)
			Ⅲ: Paclitaxel + carboplatin + bevacizumab combination only	11.2	0.908 (0.795 to 1.040, *p* = 0.16)	38.7	1.036 (0.827 to 1.297, *p* = 0.76)	
NCT00483782 **ICON7**	Newly diagnosed ovarian epithelial, fallopian tube, or primary peritoneal cavity cancer	1,528	Ⅰ: Paclitaxel + carboplatin	22.4	-	28.8	-	Perren et al. (2012)
			Ⅱ: Paclitaxel + carboplatin + bevacizumab	24.1	0.87 (0.77 to 0.99, *p* = 0.04)	36.6	0.64 (0.48 to 0.85, *p* = 0.002)	

### 2.2 Apatinib (YN968D1)

Apatinib is a new generation oral tyrosine kinase inhibitor that highly selectively targets the VEGFR2 signaling pathway, primarily blocking VEGFR-induced endothelial cell migration and proliferation and reducing tumor microvessel density (Tian et al., 2011). A phase II prospective clinical study evaluated the efficacy and safety of apatinib monotherapy in 28 patients with recurrent platinum-resistant EOC, showing an ORR and disease control rate (DCR) of 41.4% and 68.9%, respectively; a mPFS and OS of 5.1 and 14.5 months, respectively; manageable toxicity; and good patient tolerance (Miao et al., 2018). This trial provided evidence that apatinib monotherapy is effective in patients with relapsed/platinum-resistant OC. In addition, the results from several clinical trials have shown that combination therapy with apatinib is beneficial and well tolerated by patients, although fistulas may occur (Teo et al., 2015). Unlike the single-arm phase II trial AEROC (NCT02867956), APPROVE (NCT04348032) enrolled more patients (152) and added a monotherapy arm with the chemotherapeutic agent pegylated liposomal doxorubicin (PLD) (Lan et al., 2018; Wang et al., 2022). In the comparison of PLD alone and PLD in combination with apatinib, the results showed that the median OS was prolonged by 2.5 months and 8.6 months, respectively, with a favorable safety profile. This study initially showed that combining apatinib with PLD in second-line chemotherapy in patients with platinum-resistant recurrent ovarian cancer (PROC) was more effective than PLD alone (Wang et al., 2014). Wang et al. conducted a small retrospective study in which they collected and analyzed clinical data from 41 patients who relapsed after receiving apatinib monotherapy or apatinib in combination with chemotherapy, and they found that apatinib delayed progression in OC patients with biochemical relapse (defined as only CA-125 increased by more than twice the normal value, usually 2–6 months earlier than clinical evidence, such as imaging presentation. (Wang et al., 2022). We look forward to further validating this result with a large-scale trial. Furthermore, a study identified profibronectin-1 (FBN1) as a key target of chemoresistance in OC by constructing an OC-like organ model. FBN1 regulates glycolysis and angiogenesis *via* VEGFR2/STAT2, and its inhibition reduced sensitivity to cisplatin in this model, providing evidence for the combination of an FBN1 inhibitor and apatinib for the treatment of platinum-resistant OC (Wang et al., 2022b). Yang et al. demonstrated that *in vivo*, PD-L1 binds directly to VEGFR2, induces tumor angiogenesis, and relies on the c-JUN/PD-L1/VEGFR2 signaling axis to participate in the progression, invasion, and metastasis of OC, which provides evidence for the use of the pD-L1 inhibitor durvalumab combined with the VEGFR2 inhibitor anlotinib to improve the OC therapeutic effect (Yang et al., 2021a). Based on previous studies, angiogenesis inhibitor-induced hypoxia induces HRDs by affecting homologous recombination repair (HRR)-related BRCA1, BRCA2, and RAD51, resulting in enhanced effects of PARPis (Figure 2) (Mittica et al., 2018; Ashton and Bristow, 2020). Furthermore, PARP1 inhibition impedes HIF1α accumulation and attenuates HIF1α-mediated anti-angiogenic drug resistance (Martí et al., 2021; An et al., 2021), and the PARP1 inhibitor fluzoparib in combination with anlotinib contributes to treatment efficacy (Wang et al., 2019a). We expect the results of relevant clinical experiments to provide meaningful guidance for the treatment of OC.

### 2.3 Anlotinib (AL3818)

Anlotinib is a new oral multitarget tyrosine kinase inhibitor. Anlotinib selectively targets VEGFR2/3 and fibroblast growth factor receptor (FGFR) one to four with high affinity to inhibit VEGF/VEGFR signal transduction and platelet-derived growth factor receptor α and β as well as the activity of stem cell factor receptor (c-Kit) and Ret (Sun et al., 2016). Many studies have shown that anlotinib has a good therapeutic effect in patients with platinum-resistant and refractory OC. For example, a retrospective study in 2020 showed the benefits of single-drug treatment with anlotinib; although the number of patients included in this study was small, the DCR was 85.7%, suggesting that anlotinib had good application prospects (Ni et al., 2020). Therefore, large-scale clinical prospective and retrospective studies are needed for further verification. Su et al. found that anlotinib reactivates the immune microenvironment and relies on CD4^+^ T-cell to promote the normalization of tumor blood vessels; therefore, the combination of anlotinib and ICIs can enhance treatment efficacy (Su et al., 2022). A small retrospective study involving 32 patients with advanced EOC who had received at least two existing standard treatments showed that the efficacy of anlotinib combined with a PD-1 blocker in the treatment of advanced EOC was good, with a mPFS of 6.8 months and a median OS of 18.5 months (Li et al., 2022c). Lan et al. found that the objective effective rate of erlotinib combined with the PDL1 inhibitor TQB2450 was 47.1%, the DCR was 97.1%, and the mPFS was 7.8 months, showing promising antitumor activity and controllable toxicity (Lan et al., 2022). In patients with platinum-resistant or refractory OC, a phase Ib study of the injection of the PD-L1 inhibitor TQB2450 in combination with anlotinib has preliminarily demonstrated an antitumor effect, with a duration of remission (DOR) reaching 97.1% and a DOR of more than 8 months in 61.3% of patients; hypertension and palmar-plantar erythrodysesthesia syndrome were the most common adverse events (AEs), with rates for both reaching 29.4%, and a further phase III experiment (NCT05145218) is recruiting patients (Lan et al., 2022).

### 2.4 Cediranib (AZD2171)

Cediranib is an oral small-molecule multitarget tyrosine kinase inhibitor that targets VEGFR-1, VEGFR-2, VEGFR-3 and c-kit that has shown antitumor activity against recurrent EOC (Matulonis et al., 2009). Matulonis et al. conducted a phase II study of cediranib monotherapy in patients with recurrent OC and obtained an overall remission rate of 17% and a mPFS of 5.2 months; major adverse effects included grade 3 hypertension (46%), fatigue (24%), diarrhea (13%), and grade 2 hypothyroidism (56%) but no intestinal perforation or fistulas (Ledermann et al., 2021). Compared with previous studies on bevacizumab monotherapy, the advantage is that when PFS is prolonged, the incidence of intestinal perforation or fistula treated with cediranib monotherapy is lower (Cannistra et al., 2007; Burger et al., 2007). It is worth noting that, on the one hand, cediranib targets vascular endothelial growth factor receptor (VEGFR) to induce HRD inhibition related to the hypoxic microenvironment, including the downregulation of HRR protein expression (Lu et al., 2011; Lim et al., 2014); on the other hand, cediranib inhibits platelet-derived growth factor receptor (PDGFR) and activates protein phosphatase 2A (PP2A), which mediates the inhibition of HRDs unrelated to hypoxia (Kaplan et al., 2019). Therefore, a series of clinical experiments were carried out on cediranib in combination with PARPis. ICON6 and ICON9 are phase III clinical trials for patients with PSROC. As of 2016, the mPFS in ICON6 in the chemotherapy plus cediranib and placebo maintenance therapy groups was 11.0 months and 8.7 months, respectively (*p* < 0.0001) (Ledermann et al., 2021). The ICON6 study demonstrated that cediranib maintenance therapy prolongs mPFS more effectively. However, ICON9 research focuses on the difference in the effectiveness of olaparib single-drug maintenance therapy or olaparib in combination with cediranib treatment. At present, patients are being recruited, and we look forward to the publishing of the results (Elyashiv et al., 2021). Interestingly, a preclinical study carried out by Francesca Bizzaro et al. found that for patients with OC xenotransplantation (OC-PDX), olaparib and cediranib played a synergistic role by affecting tumor cells and the TME, respectively. Regardless of the HRR mutation status, cediranib combined with olaparib shows a wider effect of inhibiting tumor vascular growth than the single drug in OC-PDX (Lheureux et al., 2020). A small study found that when the PARPi drug resistance mechanism is different, the antitumor activity of cediranib and olaparib will also change, just as their efficacy in patients with homologous recombination gene and/or ABCB1 reverse mutations is poor (Zimmer et al., 2019). Another phase I study (NCT02484404) of the combination of olaparib plus cediranib and durvalumab in patients with recurrent platinum-resistant OC found this combination to be tolerable and initially active; thus, the study has moved into a second phase of enrolling more patients with recurrent OC (Zimmer et al., 2019). We expect more practical strategies for the posterior-line treatment of patients with recurrent OC.

## 3 Targeting the cell cycle and DNA damage repair

### 3.1 Poly (ADP-ribose) polymerase (PARP)

**FIGURE 3:**
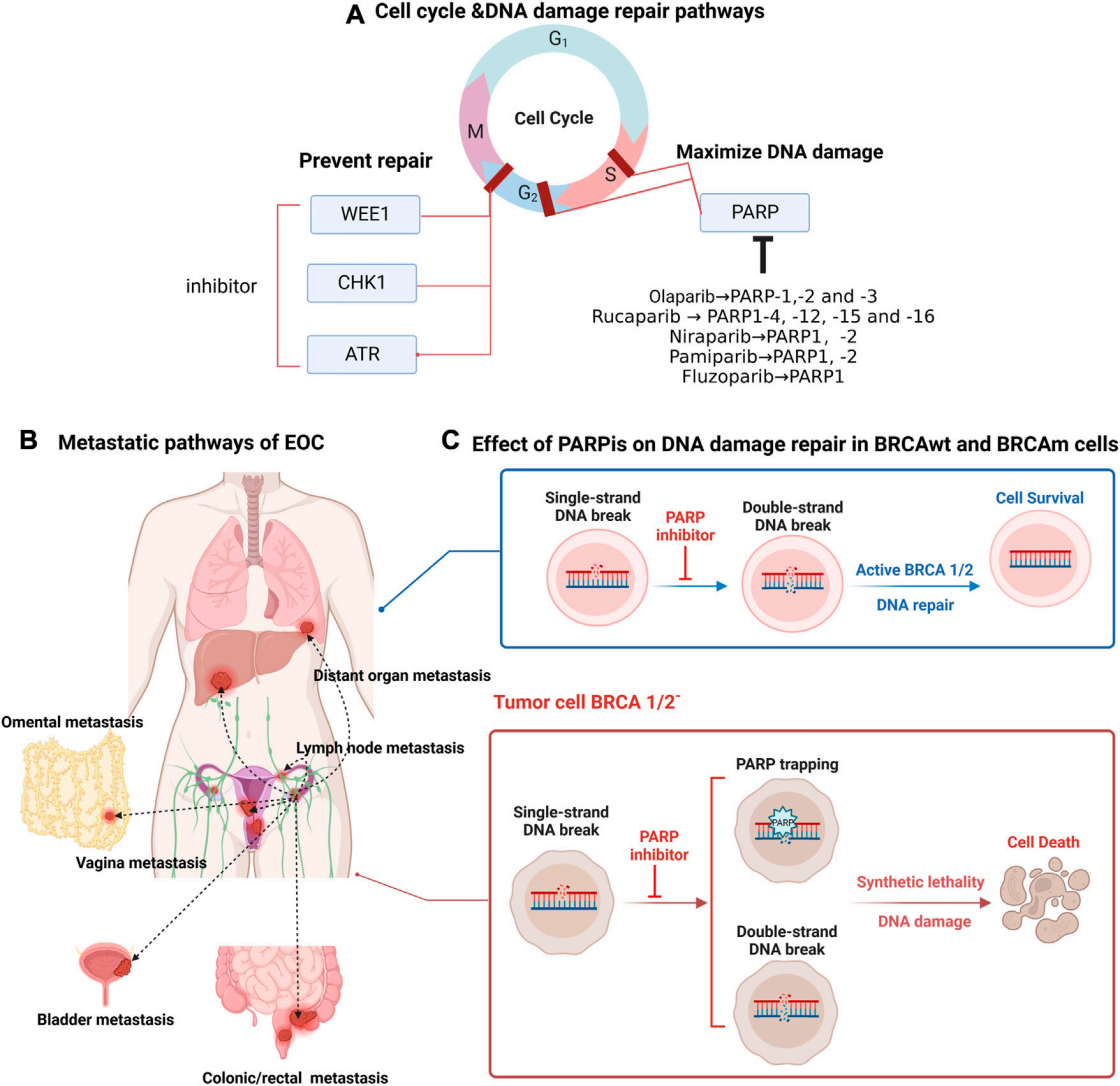
Targeting ovarian cancer cell cycle and DNA damage repair pathways, distant metastatic sites in ovarian cancer, principles of action of PARP inhibitors. **(A)** PARP proteins are involved in S-phase and G2-phase repair of the cell cycle. PARPis amplify DNA damage, and the common types of PARPis and their acting PARP proteins are described here; the main loci involved in G2-phase repair include WEE1, CHK1 and ATR, and the design of corresponding inhibitors can help prevent DNA damage repair. **(B)** Common metastatic pathways in epithelial ovarian cancer include: direct invasion of adjacent organs (vagina, bladder, rectum/colon, contralateral ovary); implantation metastases in the omentum and abdominal cavity; lymphatic metastases and hematogenous metastases involving distant organs. **(C)** Poly (ADP-ribose) polymerase (PARP) protein recognizes and repairs DNA single-strand breaks (SSBs), and unrepaired SSBs are converted to double-strand breaks (DSBs) with PARPi, which relies on the homologous recombination repair pathway for cell survival; in the presence of homologous recombination defects, including BRCA1/2 mutations, double-strand breaks cannot be repaired, causing cell death.

Poly (ADP-ribose) polymerase (PARP) is a key factor involved in DNA damage repair; on the one hand, PARP is involved in DNA single-strand break (SSB) repair-dependent base excision repair (BER) (Figure 3) (Banerjee et al., 2021); on the other hand, in double-strand break (DSB) repair, PARP contributes to HRR and inhibits error-prone non-homologous and microhomologous-mediated end-joining repair (Mirza et al., 2018). By competitively binding to NAD^+^, PARPis interfere with BER and inhibit PARP protein activity to prevent or slow down replication divergence, ultimately leading to SSB to DSB progression (Dziadkowiec et al., 2016). In addition, PARPis promote the capture of PARP proteins at the site of DNA damage, leading to a sustained S phase in cells, and the captured PARP-DNA complexes have been shown to be more cytotoxic than unrepaired SSBs (Murai et al., 2012; Gourley et al., 2019). Thus, PARPis enable error-prone repair processes to dominate and exert synthetic lethal effects in cells accompanied by mutations in HRR-associated genes. Many PARPis, such as olaparib, rucaparib, niraparib, pamiparib (BGB-290), and fuzuloparib, play an irreplaceable role in the first-line maintenance treatment and second-line and beyond posttreatment of OC.

#### 3.1.1 Olaparib

##### 3.1.1.1 First-line treatment with olaparib

Olaparib was the first approved PARPi (Figure 1) and has a strong inhibitory effect on PARP enzymes (including PARP-1, PARP-2 and PARP-3) (Figure 3). In SOLO-1 (Table 2), after 2 years of olaparib maintenance therapy in patients with newly diagnosed BRCAm advanced OC, a 5-year follow-up study (through 5 March 2020) showed an mPFS of 56.0 months in the olaparib group compared with 13.8 months in the placebo group. Maintenance therapy with olaparib for 2 years extended PFS to as long as 4.5 years, and the results of this study support the use of olaparib maintenance therapy as the standard of care for this group of patients (Banerjee et al., 2021). The latest OS data from the SOLO-1 study were updated at the European Society for Medical Oncology (ESMO) 2022 meeting, where it was reported that the longest OS data to date had been obtained with olaparib (7-year follow-up showing that 67.0% and 46.5% of patients in the olaparib group and the placebo group survived, respectively); however, no new safety events were identified. The median OS endpoint remained unmet, with a high OS of 75.2 months in the placebo group (DiSilvestro et al., 2023). On the basis of the data reported thus far, it is not difficult to speculate that the survival benefit from the administration of 2 years of olaparib maintenance therapy persists for several years after the end of treatment, with long-term survival truly being achieved.

**TABLE 2:** Summary of clinical trials of parmiparib in the treatment of ovarian cancer.

Number	Combination agent	Population	N	Phase	Status	Primary outcome measures/results
NCT03333915	NR	Chinese patients with advanced OC, fallopian cancer, and primary peritoneal cancer	128	I/II	Active, not recruiting	• Phase I: Number of participants with treatment-related adverse events
						• Phase II: ORR
NCT02361723	NR	PSOC with known or suspected harmful g/s BRCAm or HRD (+)	101	IA/I B	Completed	• ORR: CR + PR
						• Primary PK 1/PK 2/PK 3
NCT03519230	NR	Chinese patients with PSOC	216	Ⅲ	Active, not recruiting	• PFS
NCT05489926	NR	Patients with EOC who had previously been treated with a PARP inhibitor	15	Ⅱ	Recruiting	• CBR: CR + PR
NCT03933761	NR	Patients with HGSOC or carcinosarcoma with fusion positive and reverse negative BRCA1/2 m	0	Ⅱ	Withdrawn	• CBR as assessed by RECIST v1.1 or by Gynecological Cancer Intergroup (GCIG) CA125 criteria
NCT05494580	Surufatinib	PROC patients who have received PARP inhibitor treatment once	38	Ib/II	Not yet recruiting	• MTD (phase Ib)
						• Determination of PR2D (phase Ib)
						• ORR (phase II): CR + PR
NCT04985721	Tislelizumab	Patients with BRCA1/2m or without BRCA1/2m but with other germline or somatic mutations in other HR genes	60	Ⅱ	Recruiting	• CBR: PR + CR
**NCT05044871**	**NR**	**Patients with PROC**	**160**	**Ⅱ**	**Not yet recruiting**	• **ORR: CR + PR**

This study is an open-label, multicenter, umbrella study aimed to evaluate the combined, biomarker-driven, targeted treatment efficiency of Pamiparib, Bevacizumab, Tislelizumab, and Nab-paclitaxel in patients with platinum-resistant recurrent ovarian cancer (PROC). NCT05044871 is the NCT number of this study. NR indicates no combination of drugs. Patients with PROC shows that this study recruit patients with platinum-resistant recurrent ovarian cancer (PROC). ORR: CR + PR is the main clinical evaluation index of this experiment. ORR, Objective response rate; CR, complete response; PR, partial response.

The PAOLA-1 trial, with a median follow-up of 22.9 months, showed a significant PFS benefit in patients with advanced EOC by adding olaparib to bevacizumab maintenance therapy, using first-line platinum-containing agents in combination with bevacizumab, when compared to placebo maintenance therapy (22.1 months vs 16.6 months, *p* < 0.001) (Ray-Coquard et al., 2019). The study further stratified patients according to HRD status and whether BRCA was mutated and found that bevacizumab combined with olaparib was beneficial regardless of BRCA mutation status as long as the tumor was positive for HRD (Ray-Coquard et al., 2019); in either higher risk (stage III, prior surgery and residual disease or neoadjuvant chemotherapy (NACT); stage IV) or lower risk (stage III, prior surgery, no residual disease) patients, olaparib maintenance regimen can be beneficial (Harter et al., 2022). Interestingly, Callens et al. designed the tBRCA assay based on the findings of this study, and they found that the tBRCA assay more reliably identified the population that could benefit in the clinic than germline (gBRCA) assays (Callens et al., 2021). A 2021 study that jointly analyzed data from patients with BRCA mutations in SOLO1 and PAOLA-1 compared newly diagnosed BRCA-mutated OC PFS improvement in patients. Olaparib in combination with bevacizumab for first-line maintenance was the best and more appropriate for patients with BRCA-mutation or HRD-positive OC (Vergote et al., 2021). Updated secondary PFS (PFS2) data at a median follow-up of 35.5 and 36.5 months for PAOLA-1 in 2022 showed that bevacizumab monotherapy in combination with olaparib vs the combination placebo group had a mPFS2 of 36.5 and 32.6 months, respectively, and the effective improvement in PFS2 suggests that the combination regimen provided sustained benefit even after progression with the first treatment (González-Martín et al., 2022).

##### 3.1.1.2 Second-line and beyond treatment with olaparib

In SOLO-2, a study in 2021 updated the median OS prolongation by 12.9 months when reaching a median follow-up time of more than 5 years based on the previously reported significant prolongation of the mPFS in the olaparib group compared to the placebo group (Hutchinson, 2017; Francis et al., 2022), and the olaparib maintenance phase would not have a negative impact on health-related quality of life (HRQOL) (Friedlander et al., 2018); this study supports the benefit of maintenance treatment with olaparib in patients with PSROC with BRCA1/2 mutations. Based on data provided by SOLO2, Frenel et al. evaluated the time to second progression (TTSP) from RECIST progression to the next progression/death in placebo-treated and olaparib-treated cohorts of patients who received non-platinum and platinum-based chemotherapy, respectively, and they found that when second-line olaparib was maintained for reprogression, patients with recurrent BRCA1/2-mutant PSROC had weaker efficacy when platinum-containing chemotherapy was reapplied than patients who had not previously used PARPis (Frenel et al., 2022). Francis et al. found that dose changes within the first 12 weeks of treatment did not impact survival outcomes, suggesting that in clinical practice, patients who had olaparib reduced or even discontinued due to AE intolerance would not experience an impact on PFS and OS (Domchek et al., 2016). An updated median OS of 32.7 months at a median follow-up of 33.1 months for the phase IIIb OPINION study was published at the 2022 ESMO Annual Meeting; the Kaplan-Meier analysis showed OS rates of 65.8% and 54.9% at 24 and 30 months, respectively, and these data further support the use of olaparib maintenance therapy for the treatment of non-gBRCAm PSROC (Poveda Velasco et al., 2022). The L-MOCA study was the first clinical study to assess the efficacy and tolerability of olaparib maintenance treatment in Asian PSROC patients, and an mPFS of 16.1 months for all patients as of 25 December 2020, was reported (Gao et al., 2022). Subgroup analysis showed that compared to the corresponding wild-type group mPFS, the BRCA mutation group mPFS (21.2 months vs 11.0 months) and the HRR mutation group mPFS (18.3 months vs 13.3 months) were better. The AE incidence was 99.1%, with the most common AE being anemia (76.4%), and 9.4% of patients discontinued treatment due to treatment-related AEs. This study showed that in Asian PSROC patients, olaparib maintenance therapy had significant efficacy regardless of BRCA status and was well tolerated by patients (Gao et al., 2022). In 2014, the Food and Drug Administration (FDA) approved olaparib in this population based on the results of Study 42 (NCT01078662), in which patients with recurrent gBRCAm OC who had received at least three chemotherapy regimens responded durably to olaparib (Figure 1) (Domchek et al., 2016). In 2020, according to data reported in the SOLO3 trial (NCT02282020), for PSROC patients with gBRCAm and ≥2 prior lines of platinum-based chemotherapy, olaparib monotherapy showed clinically relevant and significant improvements in ORR (primary endpoint) and PFS (secondary endpoint) compared with single-agent non-platinum-based chemotherapy, and the differences were statistically significance (Penson et al., 2020). At the American Society of Gynecologic Oncology (SGO) 2022, a recent analysis of the SOLO3 trial showed that the olaparib group outperformed the non-platinum chemotherapy group in PFS2, with similar OS in both treatment groups and no new safety signals identified; this provides support for olaparib as a platinum-free chemotherapy treatment strategy for patients with PSROC in the third line and beyond (Penson et al., 2022). All the studies provide convincing evidence for the use of olaparib in the second-line and beyond treatment of OC.

#### 3.1.2 Rucaparib

##### 3.1.2.1 First-line treatment with rucaparib

Rucaparib inhibits PARP1-4, −12, −15 and −16, as well as tankyrase 1 and 2 (Figure 3) (Musella et al., 2018). Updated ATHENA-MONO results showed a significant improvement in PFS for all patients studied in the rucaparib group (intention to treat (ITT) patients or all patients) (9.2 months vs 12.1 months); improved mPFS in the HRD-positive patient group (11.3 months vs 28.7 months, *p* = 0.0004); and a treatment benefit at the endpoint of PFS in the HRD-negative subgroup (9.1 months vs 12.1 month, *p* = 0.0284). The incidence of myelodysplastic syndrome (MDS)/acute myeloid leukemia (AML) in the rucaparib group while on treatment was 0.2% (González-Martín et al., 2019). This study supports the significant benefit of rucaparib monotherapy as first-line maintenance for OC, regardless of HRD status, in patients with advanced OC.

##### 3.1.2.2 Second-line and beyond treatment with rucaparib

ARIEL2 (NCT01891344) showed a better outcome for patients with BRCAmut high-grade ovarian cancer (HGOC) who had received at least two chemotherapies, with an mPFS of 7.8 months, an ORR of 45.7%, and a DOR of 9.2 months, than in patients with BRCAwt/LOH-high and BRCAwt/LOH-low HGOC (Swisher et al., 2021b). Based on the phase II study, olaparib and niraparib alone have also been approved by the FDA for the third-line treatment of recurrent OC (Figure 1). In addition, ARIEL2 subgroup analysis showed longer PFS in patients with loss of heterozygosity (LOH)-high platinum-sensitive HGOC than in patients with LOH-low cancer among BRCAwt patients, suggesting that the assessment of tumor LOH may be a useful approach to identify patients with BRCA wild-type platinum-sensitive OC (Swisher et al., 2021a; Swisher et al., 2017). The ARIEL3 study showed that, in addition to the effect on patients with PSROC, patients who had received at least two platinum-based chemotherapies showed a significantly improved PFS, with a median follow-up time of 28.1 months, compared with the placebo group. The chemotherapy-free interval (CFI), time to start first subsequent treatment (TFST), time to disease progression on subsequent treatment or time to death, and the time to start second follow-up treatment (TSST) were all statistically significantly delayed in the rucaparib maintenance group compared with the intention-to-treat, BRCAm and homologous recombination deficient cohort (PFS2), and the updated safety data are consistent with previous reports (Ledermann et al., 2020; Clamp et al., 2021; Tomao et al., 2020; O'Malley et al., 2022). This suggests that maintenance treatment with rucaparib significantly delays the start of follow-up treatment. The ARIEL4 study showed that PFS was effectively prolonged by a median follow-up time of 25.0 months in the rucaparin group compared to the chemotherapy group; this result supports the use of rucaparib in patients with recurrent BRCA1/2-mutated OC as an alternative to platinum-based chemotherapy (Kristeleit et al., 2022; O'Donnell, 2022).

#### 3.1.3 Niraparib

##### 3.1.3.1 First-line treatment with niraparib

Niraparib is a highly effective and selective small-molecule PARP 1/2 inhibitor (Figure 3) (Jones et al., 2015). PRIMA demonstrated for the first time that niraparib single-agent first-line maintenance therapy was effective in prolonging PFS, with a 2-year OS rate of 84% and a 38% reduction in the risk of recurrence or death, when used after platinum-containing chemotherapy for advanced OC, regardless of BRCA mutation/HRD status (O'Cearbhaill et al., 2022). The PRIME study is the largest randomized controlled phase III clinical study of a PARPi for first-line maintenance therapy in patients with advanced OC in China; data published in the Chinese population complement the PRIMA findings (Li et al., 2022a). At SGO 2022, the updated PRIME study results were encouraging, with niraparib single-agent maintenance prolonging the mPFS to 14 months in patients with “double-negative” (advanced newly diagnosed BRCA and HRD negative) OC, completely rewriting the prognosis for the “double-negative” population (Del Campo et al., 2019). In addition, the PRIME study used a personalized starting dose, which resulted in a much lower incidence of adverse reactions and better patient compliance in the niraparib group than in the PRIMA study (Del Campo et al., 2019). At SGO 2022, the latest data from the OVARIO trial were updated with sequential niraparib combined with bevacizumab maintenance therapy after first-line platinum-containing chemotherapy combined with bevacizumab in patients with newly diagnosed stage IIIB to IV OC, with an mPFS of 19.6 months at 28.7 months, respectively; immature OS data; and an OS event rate of 23.8%. This single-arm study demonstrated that first-line maintenance therapy with niraparib in combination with bevacizumab results in promising PFS outcomes regardless of the patient’s biomarker profile (Hardesty et al., 2022).

##### 3.1.3.2 Second-line and beyond treatment with niraparib

The NOVA study included for the first time a full population of patients with PSROC exploring niraparib maintenance therapy, regardless of gBRCA mutation status, and its primary PFS endpoint was significantly prolonged in the niraparib-treated group compared with the placebo maintenance therapy group (gBRCA mutation population: 21.0 months vs 5.5 months; non-gBRCA mutation population: 9.3 months vs 3.9 months) (Mirza et al., 2016). Patients benefited from niraparib maintenance therapy regardless of whether the response to the last platinum-based treatment was a partial response or a complete response (Mirza et al., 2020). In the latest long-term follow-up data presented at SGO 2021, in the gBRCA mutation population, there was a 34% reduction in the risk of death and a 9.7-month increase in median OS in the niraparib group compared to the placebo group (43.8 months vs 34.1 months). However, in the non-gBRCA mutation population, there was no significant difference in OS between the niraparib and placebo groups (Matulonis et al., 2021). In addition, long-term safety data showed that hematological adverse reactions to niraparib occurred mainly in the first year of dosing and then decreased year by year, supporting that niraparib can be used for long-term maintenance therapy in patients with OC (Mirza et al., 2020). In a retrospective analysis of the NOVA study, it was found that patient weight should be considered when initiating niraparib treatment and that dose reduction (from 300 mg per day to 200 mg per day) in patients in the low weight group significantly reduced complications without compromising efficacy (Berek et al., 2018).

The QUADRA study showed that a 28% ORR was reached in the primary study population (patients with advanced HRD-positive PSROC in treatment lines 4–5), with OS reaching 26 months, 19 months, and 16.6 months in patients with BRCA-mutant HRD-positive, HRD-negative, or unknown HRD status, respectively, and 17.2 months in all patients in treatment lines 4 and above. In addition, in patients with platinum-sensitive OC, the ORR was 39% and 26% in those with BRCA-mutant and HRD-positive tumors, respectively. In patients with platinum-resistant and platinum-refractory OC, the ORR was 27% and 10%, respectively (Moore et al., 2019). The QUADRA study demonstrated that niraparib monotherapy prolonged OS in patients with platinum-resistant or refractory OC treated with third-line chemotherapy and beyond (Moore et al., 2019); this prompted the FDA to expand the indications for receiving niraparib monotherapy to patients with BRCA-mutant HRD + tumors for the first time, offering hope to more patients (Figure 1).

#### 3.1.4 Pamiparib (BGB-290)

Pamiparib is a potential selective oral PARP1/2 inhibitor independently developed in China (Figure 3). Preclinical models have shown that pamiparib has pharmacological properties such as blood‒brain barrier penetration and PARP-DNA complex capture (Xiong et al., 2020). Parmiparib is not a substrate of P-glycoprotein (P-gp) or breakthrough cancer resistance protein (BCRP); thus, it is expected to overcome the PARPi resistance problem caused by their overexpression (Durmus et al., 2015). In a key phase II clinical trial (NCT0333915), pamiparib monotherapy showed sustained antitumor activity and controllable safety in patients with gBRCA-mutated OC who had previously received at least two lines of chemotherapy (Wu et al., 2022). At ESMO 2020, Wu presented results from his phase II data showing that parmiparib showed significant clinical benefit in both PSROC and PROC patients; ORRs were 64.6% and 31.6%, respectively. Notably, Wu’s team also concluded that parmiparib is expected to usher in a new era of platinum-free chemotherapy treatment for OC patients (Lickliter et al., 2022). On 7 May 2021, the State Drug Administration (National Medical Products Administration (NMPA)) of China approved the marketing of pamiparib capsules for the treatment of patients with recurrent advanced OC, fallopian tube cancer or primary peritoneal cancer with gBRCA mutations who have been previously treated with second-line or beyond chemotherapy (Figure 1). Given the promising application of pamiparib in OC, in this review, we summarize the findings of all pamiparib clinical trials (https://clinicaltrials.gov) (Table 2).

#### 3.1.5 Fuzuloparib

The FZOCUS-2 study showed a significant improvement in the mPFS in the fuzuloparib group compared to the placebo group in the overall PSROC population. Subgroup analysis showed a direction of benefit consistent with that of the overall population regardless of the presence of gBRCA 1/2 mutations. Based on this study, the first indication for fuzuloparib in the treatment of OC was approved (Li et al., 2022b).

The results of the FZOCUS-3 study showed that in patients with PSROC with gBRCA1/2 mutations previously treated with second-line or beyond chemotherapy, fuzuloparib had an objective remission rate (ORR) of 69.9%, a median time to remission (mDOR) of 10.2 months, and an mPFS of 12.0 months, with safe and controlled treatment and only one AE-induced treatment discontinuation (0.9%) (Li et al., 2021). Based on this study, the second indication for fuzuloparib in the treatment of platinum-sensitive recurrent ovarian, fallopian tube, or primary peritoneal cancer with gBRCAm after prior second-line chemotherapy or higher was approved.

### 3.2 Targeting folic acid receptor α (FR-α)

Folate receptor α (FRα) is a cell surface transmembrane glycoprotein whose main role is to transport folate to promote cell proliferation and DNA synthesis, and its overexpression is closely associated with an increased metabolic demand for single carbon units in tumor cells. FRα is also involved in cancer cell division and migration, and the inhibition of this receptor provides a degree of direct anticancer activity (Scaranti et al., 2020). Because the percentage of EOC tumors with FRα overexpression is close to 80%, targeting FRα has become a promising treatment for EOC (O'Malley et al., 2020; Köbel et al., 2014).

Mirvetuximab soravtansine (IMGN853, MIRV), a first-in-class ADC consisting of a folate receptor alpha-binding antibody, a cleavable linker, and the maytansinoid payload DM4 (a potent microtubulin-targeting agent), causes cycle arrest and apoptosis in targeted cancer cells, and the drug has shown promising activity in women with platinum-based chemoresistant OC (Ponte et al., 2016). At the 2021 ASCO Annual Meeting, partial FORWARD II trial results were reported, and the investigators found strong antitumor activity and tolerability of MIRV in combination with bevacizumab in patients with FRα (+) OC with unknown platinum sensitivity. Based on previously reported data on MIRV/BEV in patients with platinum-resistant OC, this study suggests that MIRV + bevacizumab has the potential to be the combination of choice for patients with FRα-high expressing recurrent OC, regardless of platinum sensitivity (Lheureux et al., 2019a; Sisay and Edessa, 2017). The SORAYA trial enrolled a total of 106 patients with platinum-resistant OC with high FRα expression who had received up to three prior treatment regimens, at least one of which included bevacizumab. First-line data from SORAYA were presented at the 2022 SGO Annual Meeting, and an ORR of 32.4% and a median DOR of 6.9 months for the overall efficacy population were reported. Eighty-six percent of patients experienced all-grade AEs. Most AEs were of lower grade, with donor keratopathy and blurred vision, occurring in 47% of patients, being labeled as mirvetuximab sorafenib-specific AEs (SGO, 2022; Zamarin et al., 2020). In another phase III study, benefits of MIRV treatment compared to chemotherapy were demonstrated in terms of improvements in the secondary study endpoints of the ORR, CA-125 and patient-reported outcomes, and MIRV demonstrated a more manageable safety profile (Moore et al., 2021). In 2022, MIRV was approved by the FDA for application in the treatment of (FR α)-positive, platinum-resistant EOC (Figure 1).

## 4 Targeting the tumor immune signaling pathway

### 4.1 Immunosuppressive TME of OC

**FIGURE 4:**
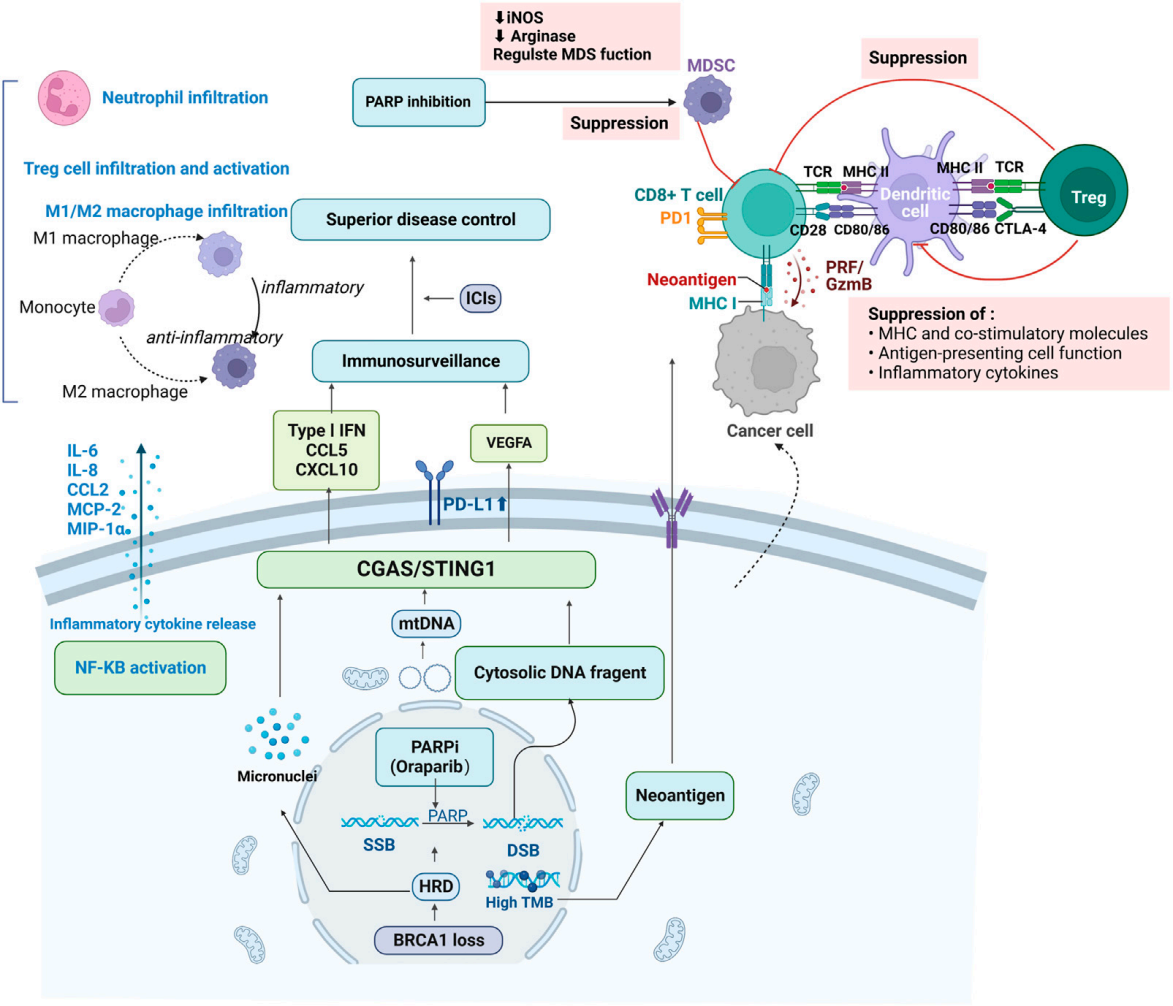
Synergy of immune checkpoint inhibitors with PARPi and tumor suppressive immune microenvironment. PARPis induces double-strand breaks in HRD cells, generating cytoplasmic dsDNA fragments, micronuclei and mtDNA, which trigger the activation of the STING pathway by binding to cyclic GMP-AMP synthase (cGAS); on the one hand, it upregulates the secretion of type I interferon, CCL5, CXCL10 and VEGFA, promoting immune escape. On the other hand, low levels of DNA damage stimulate infiltration of suppressive immune cells, such as myeloid-derived suppressor cells (MDSC) or tumor-associated macrophage (TAM), which leads to the release of free radicals and triggers further DNA damage. Antigen-presenting cells, including tumor-associated dendritic cells, are recruited and activated to drive STING-dependent type I IFN signaling. Increased expression of T cell-associated chemokines activates CD4^+^ and CD8^+^ T lymphocytes in tumor and bone marrow-derived cells, increasing infiltration of cytotoxic T-cell in the microenvironment and promoting reprogramming of immune cells to phenotypes with antitumor activity. PARPis also activates PD-L1 transcription, PD-1/PD-L1 blockade and enhances antitumor immunity; activation of the κB pathway and release of various cytokines inhibit epithelial-mesenchymal transition (EMT), thereby increasing immune cell infiltration at tumor sites.

Important processes of antitumor immunity include adaptive and natural immunity, which rely mainly on the recognition of tumor-associated antigens (TAAs) and tumor-specific antigens (TSAs) by immune cells (Figure 4) (Gajewski et al., 2013). OC tumors are immunogenic, and non-spontaneous antitumor immune responses can be detected in the tumors, peripheral blood and ascites of patients with EOCs (Morand et al., 2021). The histological marker of OC tumor immune recognition is tumor infiltrating lymphocytes (TILs) (Duraiswamy et al., 2021). The presence of CD3^+^ and CD8^+^ TILs in the OC TME has been demonstrated, and their recruitment is associated with a good prognosis in patients with EOC (Zhang et al., 2003; Santoiemma et al., 2016). Notably, the response rate of OC to ICIs remains suboptimal, with only 10%–15% of patients treated with single-agent ICIs showing good clinical outcomes (Chambers et al., 2021).

The main reason for this suboptimal response rate is that solid tumors, including OC, have a remodeling effect on the tumor immune microenvironment (TIM) (Figure 4) (Yang et al., 2022). On the one hand, tumor cells can alter the degree of infiltration as well as the phenotype and function of the TILs present in primary or metastatic tumor tissues directly or through the TME, leading to immune escape (Rådestad et al., 2018). On the other hand, by altering the expression of immune checkpoint-associated proteins, the activity of effector T-cell is suppressed, and the tumor-associated macrophage (TAM) phenotype is induced to convert from an inflammation-inducing M1 type to an anti-inflammatory M2 type. Thus, the efficacy of immunotherapy is closely related to the inflammatory status of the tumor site (An and Yang, 2020). Single-cell transcriptomic analysis of OC ascites and tumors confirmed significant differences in the composition and phenotype of immune cells and immunosuppressive cells in the liquid and solid TME (Zhang et al., 2018; Izar et al., 2020). According to Daniel S. Chen et al., the human TIM is grouped into three main phenotypes: inflammatory tumors, which are hot tumors in which many inflammatory cells and inflammatory factors represented by T-cell infiltrate the tumor parenchyma; immune death tumors, which are cold tumors in which there is a lack of T-cell infiltration in the tumor parenchyma or stroma; and immune rejection immune cells in the stroma, which surround the cancer nest with abundant characteristic immune cells but do not penetrate the tumor parenchyma (Hegde and Chen, 2020). For example, platinum-resistant OC that progresses within 6 months after platinum therapy exhibits a series of “cold tumor” features, namely, low infiltration of CD8 T-cell (Mariya et al., 2014) but increased activation of CD4 T-cell, increased infiltration of regulatory T-cell (Tregs) (Hao et al., 2018) and increased infiltration of PD-L1 cells (Hamanishi et al., 2015), known to promote peritoneal dissemination (Abiko et al., 2013), in which tumor cells are in an immunosuppressive microenvironment with enhanced proliferation and migration.

### 4.2 Immune checkpoint inhibitors

Based on the immunosuppressive microenvironment of OC and the background of poor responsiveness to ICIs alone, this review focuses on the combination of ICIs with other therapeutic approaches (Table 3). The combination of ICIs with different sites of action demonstrated some therapeutic efficacy.

**TABLE 3:** Summary of all clinical trials of immune checkpoint inhibitors in the treatment of recurrent ovarian cancer published in Clinical Trials.gov.

Immune checkpoint	Inhibitor	Study	Combination agent	N	Phase	Status	Reaction to platinum
PD-1	Cemiplimab	NCT04590326	±REGN5668 (MUC16xCD28, a costimulatory bispecific) or REGN4018 (MUC16xCD3)	37	I/Ⅱ	Recruiting	-
		NCT03564340	±REGN4018 (a MUC16xCD3 bispecific antibody)	554	I/Ⅱ	Recruiting	-
	Pembrolizumab (MK-3475-100/KEYNOTE-100/Keytruda)	NCT03732950	NR	30	Ⅱ	Recruiting	-
		NCT03734692	Cisplatin + rintatolimod (intraperitoneal)	45	I/Ⅱ	Recruiting	Sensitive
		NCT02674061	NR	376	Ⅱ	Completed	-
		NCT04519151	Lenvatinib	24	Ⅱ	Not yet recruiting	Sensitive
		NCT04713514	± OSE2101	180	Ⅱ	Recruiting	Sensitive
		NCT05231122	Bevacizumab ± anti-CD40 agonist monoclonal antibody CDX-1140	80	Ⅱ	Not yet recruiting	Sensitive
		NCT04361370	Olaparib + bevacizumab	44	Ⅱ	Enrolling by invitation	Sensitive
		NCT05158062	Bevacizumab + platinum-based chemotherapy (PBC)	35	Ⅱ	Recruiting	Sensitive
			Olaparib as a maintenance therapy				
		NCT05116189	paclitaxel ± bevacizumab/placebo + paclitaxel ± bevacizumab	616	Ⅲ	Recruiting	Resistant
		NCT02901899	Guadecitabine	45	Ⅱ	Active, not recruiting	Resistant
		NCT04387227	Carboplatin	22	Ⅱ	Recruiting	-
		NCT03602586	Epacadostat	14	Ⅱ	Terminated	-
		NCT04919629	APL-2 (pegcetacoplan)±bevacizumab	40	Ⅱ	Not yet recruiting	
		NCT02440425	Paclitaxel	42	Ⅱ	Completed Has Results	Resistant
		NCT02657889	Niraparib	122	Ⅰ/Ⅱ	Completed	-
		NCT02537444	Acalabrutinib (ACP-196) ± pembrolizumab	78	Ⅱ	Completed	-
		NCT03029598	Carboplatin	29	Ⅰ/Ⅱ	Completed	Resistant
		NCT02853318	Bevacizumab + cyclophosphamide	40	Ⅱ	Completed	-
		NCT04781088	Paclitaxel + lenvatinib	38	Ⅱ	Suspended	Resistant
		NCT03428802	NR	40	Ⅱ	Recruiting	-
		NCT02608684	Cisplatin + gemcitabine	21	Ⅱ	Completed	Resistant
		NCT05467670	ALX148 + Doxorubicin (PLD)	31	Ⅱ	Not yet recruiting	Resistant
		NCT03539328	Pegylated liposomal + doxorubicin/paclitaxel/gemcitabine	138	Ⅱ	Unknown	Resistant
		NCT03113487	Modified vaccinia virus Ankara vaccine expressing p53	29	Ⅱ	Active, not recruiting	-
		NCT04575961	Platinum-based chemotherapy (carboplatin + gemcitabine/carboplatin + pegylated liposomal doxorubicin)	33	Ⅱ	Recruiting	Sensitive
	Nivolumab (Opdivo)	NCT02737787	WT1/ESO-1 vaccine	11	Ⅰ	Active, not recruiting	-
		NCT02498600	±Ipilimumab	100	Ⅱ	Active, not recruiting	-
		NCT02873962	Bevacizumab/bevacizumab ± rucaparib	76	Ⅱ	Recruiting	Sensitive
		NCT03508570	±Ipilimumab	48	I b	Active, not recruiting	Resistant
		NCT05026606	Etigilimab	20	Ⅱ	Recruiting	Resistant
		NCT03100006	Oregovomab	13	I b/II a	Terminated	-
		NCT04620954	Oregovomab + PLD + carboplatin	31	I/II	Recruiting	Sensitive
		NCT04840589	ZEN003694 ± ipilimumab	36	I/I b	Recruiting	Resistant
		NCT02465060	Targeted therapy directed by genetic testing (The MATCH Screening Trial)	6,452	Ⅱ	Recruiting	-
PD-L1	Durvalumab (MEDI4736)	NCT03430518	Eribulin	9	Ⅰ	Completed	-
		NCT04742075	Olaparib + UV1	184	Ⅱ	Recruiting	-
		NCT03699449	Olaparib/chemotherapy/tremelimumab + chemotherapy/tremelimumab + paclitaxel/olaparib + cediranib	104	Ⅱ	Recruiting	Resistant
			Olaparib + cediranib (without durvalumab)				
		NCT03267589	MEDI9447 (CD73)/MEDI0562 (OX40)/MEDI0562 (OX40) + tremelimumab (without durvalumab)	25	Ⅱ	Completed	-
		NCT04019288	AVB-S6-500	19	Ⅰ/Ⅱ	Active, not recruiting	Resistant
		NCT03277482	Tremelimumab + radiotherapy	16	Ⅰ	Terminated	-
		NCT03026062	Tremelimumab	175	Ⅱ	Recruiting	Resistant
		NCT02953457	Olaparib	40	Ⅱ	Active, not recruiting	Resistant/sensitive
		NCT02431559	Motolimod + PLD	53	Ⅰ/Ⅱ	Completed	Resistant
		NCT02484404	Olaparib ± cediranib	384	Ⅰ/Ⅱ	Recruiting	-
		NCT03283943	Focal-sensitizing radiotherapy	22	Ⅰ	Unknown	Resistant
		NCT04739800	Cediranib ± olaparib/cediranib + olaparib (without Durvalumab)	164	Ⅱ	Recruiting	Resistant
		NCT04015739	Bevacizumab + olaparib	74	Ⅱ	Active, not recruiting	-
	Atezolizumab (Tecentriq)	NCT03353831	Chemotherapy + bevacizumab/chemotherapy + Bevacizumab + placebo (without atezolizumab)	550	Ⅲ	Active, not recruiting	-
		NCT03598270	Platinum-based chemotherapy followed by maintenance niraparib + placebo/platinum-based chemotherapy followed by maintenance niraparib + atezolizumab	414	Ⅲ	Active, not recruiting	Sensitive
		NCT03206047	Guadecitabine + CDX-1401 vaccine	75	I/IIb	Active, not recruiting	-
		NCT02839707	PLD + bevacizumab	444	II/III	Active, not recruiting	Resistant
		NCT03430518	Eribulin	9	Ⅰ	Completed	-
		NCT03363867	Bevacizumab + cobimetinib (ABC)	29	Ⅱ	Recruiting	Resistant
		NCT02659384	Bevacizumab + placebo/bevacizumab + acetylsalicylic acid/single-agent bevacizumab	122	Ⅱ	Active, not recruiting	Resistant
		NCT04931342	Bevacizumab (non-matched)	400	Ⅱ	Recruiting	-
		NCT02891824	Avastin + platinum-based chemotherapy/placebo + avastin + platinum-based chemotherapy		Ⅲ	Active, not recruiting	Sensitive
	Avelumab (Bavencio)	NCT03312114	SAbR	5	Ⅱ	Terminated (low accrual)	-
		NCT03704467	Carboplatin + M6620	3	Ⅰ	Completed	Resistant
		NCT03330405	Talazoparib	226	I b/II	Active, not recruiting	Sensitive
CTLA-4	Ipilimumab (Yervoy)	NCT00060372	Following allogeneic hematopoietic stem cell transplantation	21	Ⅰ	Completed	-
		NCT00039091	NR	26	Ⅰ	Terminated	
		NCT01611558	NR	40	Ⅱ	Completed	Sensitive
		NCT03449108	Autologous tumor infiltrating lymphocytes LN-145-S1	95	Ⅱ	Recruiting	Resistant
		NCT04840589	Nivolumab + BET bromodomain inhibitor ZEN-3694	36	Ⅰ	Recruiting	Resistant

In a study that included 100 patients with persistent or recurrent EOC, the efficacy and safety of nivolumab (a human lgG4 anti-PD-1 receptor blocking monoclonal antibody (mAb)) in combination with ipilimumab (a recombinant IgG1 human mAb against CTLA-4) were compared with those of nivolumab monotherapy. The results showed a longer PFS in the nivolumab + ipilimumab group than in the nivolumab group (3.9months vs 2 months). The rate of grade ≥3-related AEs was slightly higher in the nivolumab + ipilimumab group than in the nivolumab group (49%vs 33%). This result suggests that despite slightly higher toxicity, the combination regimen was associated with a higher response rate and longer mPFS than the single-agent regimen, suggesting that a large study should be conducted to better assess the efficacy and safety of the combination regimen (Zamarin et al., 2020). In another study (NCT02335918), it was observed in OC patients that varlilumab, a fully human agonist anti-CD27 mAb in combination with nivolumab, did not show toxicity beyond that of either monotherapy, and prolonged PFS was more pronounced at a ≥5% increase in tumor PD-L1 and intratumoral T-cell infiltration (Sanborn et al., 2022). High VEGF stimulates the expansion of immunosuppressive cells, including Tregs and myeloid-derived suppressor cells (MDSCs), inhibits the migration of immunoreactive T-cell to the TME and promotes their apoptosis, providing a theoretical basis for the combination of angiogenesis inhibitors and ICIs (Figure 4) (Fukumura et al., 2018). In a phase II (NCT02853318) non-randomized clinical trial, the combination of pembrolizumab with bevacizumab and oral cyclophosphamide was well tolerated, showing a clinical benefit and durable treatment response (>12 months) in 95.0% of patients with recurrent OC; this combination may represent a future treatment strategy for recurrent OC (Zsiros et al., 2021) In addition, niraparib further increases immune cell infiltration in the TIM and modulates immune activity by upregulating the activity of interferon genes and interferon pathway stimulators and PD-L1 expression on the tumor cell surface, which may make the combination of niraparib and ICI more toxic (Shen et al., 2019; Wang et al., 2019b). A single-arm phase I and II trial (NCT02657889) subgroup analysis showed that niraparib combined with pembrolizumab showed an ORR benefit regardless of platinum chemosensitivity status, prior bevacizumab treatment or tumor BRCA or HRD biomarker status (Konstantinopoulos et al., 2019). Interestingly, Appleton et al. constructed a three-dimensional spheroid culture model of OC patient origin and demonstrated that either pembrolizumab or durvalumab synergized with olaparib to reduce the viability of the *in vitro* model (Appleton et al., 2021). Platinum-based chemotherapy is known to induce T-cell proliferation and activation, suggesting that the combination of ICIs may have a synergistic effect (Fucikova et al., 2022). In a study including nine patients with recurrent platinum-resistant OC (NCT03029598), pembrolizumab combined with carboplatin was effective and well tolerated; 23 patients achieved optimal objective remission, with 10.3% in partial remission (PR) and 51.7% with stable disease (SD), in addition to 17.2% with PD (Liao et al., 2021). In OC with BRCA1/2 mutations, the tumor load is increased and TILs are increased; furthermore, PD1/PD-L1 expression is upregulated in response to multiple interferon γ, P53 and BRCA mutations, thus possibly leading to greater sensitivity to PD1/PD-1 inhibitors (Jiang et al., 2021). In patients with PROC, the study by Li et al. suggested that the ORR and mPFS of PLD combined with pembrolizumab treatment were higher than those of the respective monotherapy, but with the inclusion of 23 patients in this study, a larger study is needed for validation (Lee et al., 2020).

## 5 New therapeutic methods for lipid metabolism-related targets

The characteristic site of OC metastasis is the lipid-rich omentum, and abnormal lipid metabolism plays an important role in tumor progression and metastasis (Ladanyi et al., 2018). Many studies have focused on targeting lipid metabolism-related pathways, suggesting a series of potentially effective new strategies for OC treatment. High-grade plasmacytoid ovarian cancer (HGSOC) metastasizes mainly to fat-rich areas such as the omentum, mesentery and appendicular epidermis over the colon (Figure 3). During metastasis, adipocytes in the microenvironment are recruited by cancer cells and transformed into cancer-associated adipocytes, and adipocytes are able to reprogram OC cell metabolism (Nieman et al., 2011; Mukherjee et al., 2020). When OC cells were cocultured with human omental adipocytes, tumor tissue induced adipocyte lipolysis, releasing more free fatty acids and glycerol and thus providing energy to promote rapid tumor growth while inducing OC cell migration and promoting invasion more significantly than subcutaneous fat (Nieman et al., 2011). In addition, OC cells cocultured with adipocytes have a lipid chaperone protein, FABP4, which regulates lipolysis and is upregulated in the expression of several *in vitro* cell lines of omental metastatic tumors, including OC, and FABP4 may be an important target for the treatment of intra-abdominal metastatic tumors (Nieman et al., 2011). It has been further shown that FABP4 knockdown leads to elevated levels of 5-hydroxymethylcytosine in DNA, downregulates the genetic features associated with OC metastasis, and inhibits tumor cell activity (Mukherjee et al., 2020). The evidence that FABP4 inhibitor monotherapy significantly reduced tumor load in a homozygous *in situ* mouse model and that an FABP4 inhibitor in combination with carboplatin enhanced chemosensitivity both *in vitro* and *in vivo* suggests that FABP4 may be an important target for the treatment of intra-abdominal metastatic tumors, providing an opportunity for specific metabolic targeting of OC metastasis (Mukherjee et al., 2020).

Kosuke Hiramatsu et al. suggested that lipolysis-stimulated lipoprotein receptor (LSR), which is highly expressed in EOC metastatic lymph nodes and omentum, is regarded as a neoplastic antigen that induces very low density lipoprotein (VLDL) into EOC cells, which in turn promotes lipid uptake in EOC cells and subsequent It is associated with poor prognosis, and OS was significantly shorter in patients with high expression of human LSR (hLSR) than in those with low expression (61.7 months vs 103.3 months, *p* = 0.0322). The resulting monoclonal antibody (#1–25) designed for hLSR showed significant antitumor effects by targeting the binding of VLDL to hLSR and intracellular storage of lipid metabolites (Hiramatsu et al., 2018). Recently, Lia Tesfay et al. showed that in OC tissues, cell lines and stem cell genetic models, upregulated steroid coenzyme A desaturase (SCD1) increased monounsaturated fatty acid formation to prevent ferroptosis. Blocking SCD1 had a dual antitumor effect, depleting the endogenous membrane antioxidant CoQ10 to induce ferroptosis and enhancing the toxicity of ferroptosis inducers on the one hand and triggering apoptosis by increasing the synthesis of saturated fatty acid-rich ceramides and altering the ratio of saturated to unsaturated fatty acids on the other. The findings of this study suggest that SCD1 inhibitors combined with ferroptosis inducers may be a new strategy for the treatment of OC in the future (Tesfay et al., 2019). Through single-cell sequencing and immunohistochemistry analysis of OC and paraneoplastic tissues, Lin et al. confirmed that Stanniocalcin 1 (STC1) expression was significantly upregulated in OC, especially in peritoneal metastases. STC1 promoted lipid metabolism not only through the *in vitro* pathway by upregulating lipid-related genes such as UCP1, TOM20 and perilipin1 but also through the FOXC2/ITGB6 signaling axis in OC to promote metastasis, lipid metabolism and *in vivo* cisplatin chemoresistance, suggesting that this could be a new treatment for OC patients with cisplatin chemoresistance-targeted pathways (Lin et al., 2022). Notably, metabolic reprogramming in cancer cells relies mainly on the LPA-LPAR-Gαi2 axis to induce a pseudohypoxic response involving the Rac-NOX-ROS-HIF1α pathway, which activates EMT in OC cells, leading to a diminished glycolytic rate and glycolytic capacity. Metabolic reprogramming also induces glucose transporter protein-1 (GLUT1) and glycolytic enzyme hexokinase-2 (HKII) expression, which ultimately leads to metabolic reprogramming, a shift to aerobic glycolysis in OC cells, and tumor progression promotion. Targeted inhibition of HKII by 3-bromopyruvate (3-BP) attenuates the growth of OC xenografts and shows potential for the treatment of OC (Ha et al., 2018). OC cells secrete angiotensin II (ANGII) in a positive feedback manner, triggering the classic receptor (AGTR1) pathway and EGFR transactivation, which is considered to be an important factor in the metastasis of several cancers. Peritoneal metastasis from OC is highly dependent on the formation of multicellular spheroids (MCSs), and the activation of AGTR1 is positively correlated with MCS formation and cell migration and negatively correlated with the prognosis of OC patients; therefore, targeting AGTR1 may be a strategy to eliminate the potential for peritoneal metastasis from EOC (Zhang et al., 2019a).

## 6 Other potential therapeutic targets

### 6.1 Immunization vaccines: Autologous dendritic cell immunotherapy

EOC responds poorly to ICIs due to its immunological features, including limited tumor mutational load (TMB) and poor lymphocyte infiltration. The use of immune vaccines and lysoviral therapy is a new strategy to enhance antitumor immunity in OC. A completed phase II clinical study, SOV01 (NCT02107937), found a statistically significant improvement in PFS with the addition of autologous dendritic cell immunotherapy (DCVAC) to first-line standard chemotherapy with carboplatin and paclitaxel (Rob et al., 2022). Interestingly, the clinical benefit of DCVAC was more pronounced in OC patients than in prostate and lung cancer patients, despite an antitumor immune cycle characterized by reduced expression of T-cell-associated genes (Hensler et al., 2022). However, multiple mechanisms, including the restriction of dendritic cell (DC) migration to draining lymph nodes, the immunosuppressive tumor microenvironment (TME) in OC, and metabolic restriction of tumor-associated DC activation, may lead to limited clinical efficacy of DC vaccines (O'Neill and Pearce, 2016). In this regard, several *in vitro* and preclinical studies have provided evidence that modified DC vaccines have shown greater benefit in OC treatment (Cheng et al., 2020).

In 2018, TANYI et al. used a personalized vaccine generated by autologous DCs pulsed with oxidized autologous whole-tumor cells. After the administration of a personalized vaccine generated by autologous DCs pulsed with oxidized autologous whole-tumor cell lysate (OCDC), an increase in IFN-γ-producing T-cell responsive to DC-presented tumor antigens was detected, with a significantly higher 2-year OS in patients who responded to the vaccine than in those who did not (100% vs 25%) and with good tolerability (Tanyi et al., 2018). In addition, Wen Zhang et al. found that the immune responses triggered by DC vaccines prepared with Wilms’ tumor protein 1 (WT1) peptides in patients with advanced OC were significantly associated with a decrease in bone marrow-derived suppressor cells (*p* = 0.045) in pretreated peripheral blood, which suggests the potential therapeutic effect of such vaccines (Zhang et al., 2019a). Recently, based on NY-ESO-1 fused with SecPen and ubiquitin, Yunkai Yang and his colleagues prepared a novel DC vaccine (DC-SNU) that induced stronger and specific T-cell immunity in mice (Yang et al., 2021b). In addition, in patients with advanced OC, long-term toxicity was not observed before or after the injection of a Th1 selective IGFBP-2 N-terminus vaccine, and T-cell clones were significantly upregulated (*p* = 0.03) (Cecil et al., 2021).

### 6.2 Oncolytic virotherapy

Selective infection and direct lysis of tumor cells by an oncolytic virus (OV) leads to the release of viral particles, cytokines and other tumor cell contents, and the release of various substances triggers innate and adaptive proinflammatory immune responses against tumor cells (Cook and Chauhan, 2020). For example, the treatment of cells from patients with OC with an oncolytic adenovirus (Ad5/3-E2F-D24-hTNFa-IRES-hIL2) in isolated cultures reshaped the OC immune microenvironment, activating CD4^+^ and CD8^+^ TILs, which in turn enhanced antitumor responsiveness (Santos et al., 2020). OVs have shown efficacy in preclinical models of advanced EOC, and it is significant that the interaction of PD-1 with its ligands PD-L1 and PD-L2 leads to the suppression of T and NK cells without overlapping with OV-mediated activation pathways (van Vloten et al., 2022). In the advanced EOCID8 model, Parapoxvirus ovis (Orf virus (OrfV)) treatment promoted active recruitment of NK cells in tumor cells and in the ascites TME, stimulated a strong antitumor response, activated NK cells and further induced T-cell recruitment in the OC TME through the CXCR3 chemokine axis, prolonging mouse survival (van Vloten et al., 2022). In a recent study, Lei et al. innovatively used the human IgG family as a scaffold to construct anti-CD47 mAbs piggybacking on tumor soluble herpesvirus (oHSV). This oncolytic herpes simplex virus, which maximizes Fc receptor-mediated antitumor effects and expresses anti-CD47 antibodies to block “do not eat me” signaling, has therapeutic effects (Tian et al., 2022).

Oncolytic herpes simplex virus (HSV) treatment of mice with OC carrying a platinum resistance gene disrupts the extracellular vesicle (EV) pathway associated with cisplatin efflux, not only helping to prevent drug resistance but also promoting DNA damage to activate the immune system and innate immunity and enhance the efficacy against ICIs (Hong et al., 2021). Novel active virus-like nanoparticle (VLP) delivery vehicles are more widely distributed and more long-lasting in OC ground metastatic ascites and have been shown to help improve survival in mice with peritoneal metastases from OC (Wang et al., 2020). In Table 4, we summarize all recent clinical trials using oncolytic viruses in the treatment of OC.

**TABLE 4:** Application of oncolytic viruses in the treatment of ovarian cancer.

Virus type	Study	Virus	N	Phase	Status	Method of administration	Population
Measles virus	NCT00408590	MV-CEA virus & MV-NIS virus	37	I	Completed	Intraperitoneal	Progressive, recurrent, or refractory ovarian epithelial cancer or primary peritoneal cancer
	NCT02068794	MV-NIS infected mesenchymal stem cells	57	I/Ⅱ	Recruiting	Intraperitoneal	Recurrent ovarian cancer
	NCT02364713	MV-NIS	66	Ⅱ	Recruiting	-	Platinum-resistant ovarian, recurrent ovarian carcinoma
Adenovirus	NCT02028117	Enadenotucirev	38	I	Completed	Intraperitoneal	Platinum-resistant epithelial ovarian cancer
	NCT05180851	Recombinant L-IFN adenovirus injection	28	Ⅰ	Recruiting	Even injection of the drug solution into the tumor edge	Relapsed/refractory solid tumors
	NCT03225989	LOAd703	50	I/Ⅱ	Recruiting	Intratumoral image-guided injections	Pancreatic cancer, biliary cancer, ovarian cancer and colorectal cancer
	NCT05271318	TILT-123	15	I	Recruiting	-	Platinum-resistant or refractory ovarian cancer
	NCT00964756	Ad5.SSTR/TK.RGD	11	Ⅰ	Completed	Intravenous	Recurrent ovarian cancer
	NCT00562003	Ad5-Delta 24 RGD	26	Ⅰ	Completed	Intraperitoneal	Ovarian cancer, primary peritoneal cancer
	NCT00002960	SCH 58500 (rAd/p53)	59	Ⅰ	Completed	Single intraperitoneal instillation	Primary ovarian, fallopian tube, or peritoneal cancer
	NCT00003880	SCH 58500 (rAd/p53)	132	Ⅱ/Ⅲ	Terminated	Intraperitoneal	Newly diagnosed stage III ovarian or stage III primary peritoneal cancer with residual disease following surgery
	NCT02963831	ONCOS-102	67	Ⅰ/Ⅱ	Completed	Intraperitoneal infusion	Peritoneal disease for which prior standard chemotherapy has failed and histologically confirmed platinum-resistant or refractory epithelial ovarian cancer or colorectal cancer
Vaccinia virus							
	NCT02017678	JX-594	0	Ⅱ	Withdrawn	Intravenous	Peritoneal carcinomatosis of ovarian origin in which patients are not eligible for curative treatments
	NCT02759588	GL-ONC1	64	I/Ⅱ	Active, not recruiting	Intraperitoneal	Recurrent or refractory ovarian cancer
	NCT05281471	GL-ONC1 (Olvi-Vec)	186	Ⅲ	Recruiting	Intraperitoneal catheter infusions	Platinum-resistant/refractory ovarian cancer
	NCT05051696	H101	60	_	Recruiting	Intratumor injection	Refractory/recurrent gynecological malignancies
	NCT05061537	PF-07263689	10	Ⅰ	Active, not recruiting	Intravenous	Ovarian cancer for which all available standard-of-care therapies have been exhausted
Reolysin	NCT01199263	Pelareorep	108	Ⅱ	Completed	Intravenous	Recurrent or persistent ovarian, fallopian tube or primary peritoneal cancer

## 7 Discussion

Ovarian cancer management has changed dramatically with the introduction of targeted therapy and immunotherapy into standard-of-care therapy. For patients in whom initial tumor reduction surgery is feasible, platinum-based standard chemotherapy combined with specific marker-related targeted and immune monotherapy or combination therapy is an important strategy to prevent recurrence. However, neoadjuvant chemotherapy (NACT) followed by interval cytoreductive surgery (CRS) benefits patients if they are suspected to have stage IIIC or IV invasive EOC at the time of initial treatment, tumors are unresectable, optimal resection (R0 and R1) is not achieved, or clinical or imaging assessment indicates a perioperative risk (Cook and Chauhan, 2020; Santos et al., 2020). Genomic analysis revealed that although tumor cell evolutionary mutations were not prevented during NACT treatment, NACT treatment induced transcriptome remodeling through an upregulation of the AP-1 transcriptional network and altered gene copy number in recurrent tumors (Javellana et al., 2022). Previous studies have found that the application of platinum-containing NACT regimens may be more likely to induce cancer cell stemness, leading to platinum resistance and a shortened platinum treatment-free interval (TFIp) (Liu et al., 2020). The use of alternative platinum-based NACT regimens can help avoid platinum resistance without compromising the role of subsequent platinum-based agents in adjuvant therapy. NANT pioneered the exploration of niraparib monotherapy as an alternative to platinum as neoadjuvant therapy in patients with advanced non-R0 resectable BRCAm/HRD-positive OC (Zhou et al., 2022). According to the latest data presented at SGO 2022, CA125 decreased from week 2 of dosing, with a median lower baseline concentration of 88.5% (26.8%–99.3%) after two cycles (8 weeks in total) of treatment and an ORR of up to 75%. Thrombocytopenia was the predominant TEAE, occurring in parallel with the decline in CA125. The above data provide preliminary evidence that niraparib single-agent neoadjuvant therapy is effective and has a good safety profile (Zhou et al., 2022). Expanding the trial sample size, increasing the number of patients included, and including a control group could make the findings of similar future studies more convincing. In addition, clinicians cannot ignore the issue that OC patients treated with NACT are at extremely high risk of thromboembolic events, especially those with advanced metastatic disease, and increased screening or the use of prophylactic anticoagulation are effective means of preventing associated AEs (Basaran et al., 2021). Notably, the OV21/PETROC study provided RCT data supporting that women undergoing NACT followed by optimal tumor reduction surgery would benefit from chemotherapy with intraperitoneal injections of carboplatin, informing the choice of follow-up treatment after NACT and optimal tumor reduction surgery in the clinic (Provencher et al., 2018). According to the phase III iPocc study reported at SGO 2022, intraperitoneal administration of carboplatin was superior to intravenous administration after initial surgery regardless of residual tumor size, improving PFS in patients with OC (Fujiwara et al., 2022). In addition, several preclinical and clinical studies have focused on improving the TME in OC. Adipose tissue is a key component of the metastatic microenvironment of OC, and preferential metastasis to omental adipose tissue is an important feature of metastatic OC (Motohara et al., 2019). Statins that inhibit a key enzyme of lipid metabolism (HMG-CoA reductase) have been shown to synergistically promote apoptosis with cisplatin in OC (Göbel et al., 2020). Future focus on targeting lipid metabolism-related pathways in OC could be of great value for the treatment of omental metastatic OC.

DNA methyltransferase inhibitors (DNMTis), which are epigenetic modulators, have been shown to induce the cytoplasmic sensing double-stranded RNA (dsRNA) antiviral pathway, upregulate type I IFN (Chiappinelli et al., 2015), activate CD8 T-cell, increase the number of immune cells (Wright et al., 2016; Stone et al., 2017; Vergote et al., 2018), and synergistically downregulate programmed death ligands (PD-L1 and PD-L2) with ICIs to exert antitumor effects. In patients with recurrent chemoresistant OC, hypomethylating agents (HMAs) in combination with ICIs to increase immune signaling and improve the response to immune checkpoint blockade in OC also appear to be a viable therapeutic strategy (Chiappinelli et al., 2022). A phase II clinical trial including 35 patients with platinum-resistant OC (NCT02901899) combined guadecitabine, a second-generation HMA, with pembrolizumab, an inhibitor of PD-1 and found that 34% of patients obtained clinical benefit (Chiappinelli et al., 2015). Song et al. found that ubiquitin UBR5, a protein ligase E3, was overexpressed in human OC cells, regulating the recruitment of immunosuppressive macrophages, i.e., M2 type, to the tumor site, leading to peritoneal colonization and metastasis on the one hand and promoting cell adhesion cancer stem cell (CSC) production by controlling p53 protein levels on the other hand, suggesting that targeting UBR5 in combination with other therapeutic approaches could benefit OC patients (Song et al., 2020). Indoleamine 2,3-dioxygenase 1 (IDO1) drives tumor immunosuppression in HGSOC by depleting local tryptophan and producing kynurenine inhibition, which is responsible for the downregulation of CD8^+^ tumor infiltrating lymphocytes (TILs) (Munn et al., 2016). The IDO1 inhibitor EPA effectively blocks the Kyn pathway of Trp catabolism in patients with advanced EOC, and Kyn and changes in downstream metabolites were accompanied by an overall increase in net enrichment in IFN and MHC class I antigen processing and gene presentation pathways, which positively correlated with the proportion of activated CD8 T-cell in the TME (Munn et al., 2016; Odunsi et al., 2022). However, IDO1 blockade leads to metabolic adaptation of the ovarian TME and an increase in NAD^+^, which in turn inhibits T-cell function *via* A2a and A2b purinergic receptors, decreasing T-cell proliferation and function and thereby suppressing antitumor responses; therefore, A2a/A2b purinergic receptor blockade in combination with the IDO1 inhibitor EPA helps to improve antitumor immunity in OC patients (Odunsi et al., 2022). In addition, immunotherapy with intraperitoneal injection of autologous IFN-α, IFN-γ and monocytes was mainly used in OC, and the efficacy was enhanced by the synergistic killing of tumor cells by promoting the development of monocytes toward inflammatory-responsive M1-type macrophages, combined with standard chemotherapy (carboplatin and paclitaxel) (Green et al., 2016). We believe that focusing on the different pathways associated with OC and finding appropriate synergistic strategies are the focuses of future individualized OC treatment.

Targeted therapy and immunotherapy are revolution in ovarian cancer management. Despite the promising treatments that have been developed for cancer immunotherapy, such as immune checkpoint inhibitors, there is still a need to overcome the immunosuppressive tumor microenvironment in order to improve the efficacy of cancer immunotherapy. In the near future, we should be able to dynamically assess tumor evolution and detect reliable biomarkers to identify immunotherapy effect of ovarian cancer. In addition, the appropriate dosing and scheduling of each agent should be determined in order to minimize adverse events while maximizing benefit and outcomes.

## Glossary

BRCA: breast cancer susceptibility gene
HRD: homologous recombination repair defect
OC: ovarian cancer
EOC: epithelial ovarian cancer
mPFS: median progression-free survival
OS: overall survival
PFS: progression-free survival
NCCN: National Comprehensive Cancer Network
HRR: homologous recombination repair
PARPi: poly (ADP ribose) polymerase inhibitor
TME: tumor microenvironment
ICIs: immune checkpoint inhibitors
HIF: hypoxia inducible factor
MAP: mitogen activated protein
PSROC: platinum-sensitive recurrent OC
PROC: platinum-resistant recurrent ovarian cancer
GC: gemcitabine
PC: paclitaxel and carboplatin
DCR: disease control rate
ORR: objective response rate
PLD: pegylated liposomal doxorubicin
FBN1: profibronectin-1
FGFR: fibroblast growth factor receptor
DOR: duration of remission
AEs: adverse events
PP2A: protein phosphatase 2A
PDGFR: platelet-derived growth factor receptor
SSB: single-strand break
DSB: double-strand break
BER: base excision repair
EMSO: European Society for Medical Oncology
TTSP: time to second progression
FDA: the Food and Drug Administration
SGO: Society of Gynecologic Oncology
MDS: myelodysplastic syndrome
AML: acute myeloid leukemia
HGOC: high-grade ovarian cancer
LOH: loss of heterozygosity
CFI: chemotherapy-free interval
TFST: time to start first subsequent treatment
TSST: the time to start second follow-up treatment
BRCP: breakthrough cancer resistance protein
mDOR: median time to remission
FRα: Folate receptor α
TAAs: tumor-associated antigens
TSAs: tumor-specific antigens
TILs: tumor infiltrating lymphocytes
TIM: tumor immune microenvironment
TAM: tumor-associated macrophage
mAb: monoclonal antibody
MDSCs: myeloid-derived suppressor cells
SD: stable disease
LSD: lipoprotein receptor
SCs: serous carcinomas
SCD: steroid coenzyme A desaturase
STC1: Stanniocalcin 1
GLUT1: glucose transporter protein-1
HKII: hexokinase-2
MCSs: multicellular spheroids
TMB: tumor mutational load
DCVAC: dendritic cell immunotherapy
DC: dendritic cell
OV: oncolytic virus
HSV: herpes simplex virus
EV: extracellular vesicle
VLP: virus-like nanoparticle
NACT: neoadjuvant chemotherapy
TFIp: platinum treatment-free interval
TEAE: treatment emergent adverse events
HMAs: hypomethylating agents
CSC: cancer stem cell
MSI-H: MicroSatellite Instability-High
dMMR: different Mismatch Repair
